# Expert insights on Hodgkin’s lymphoma development in an activated PI3K delta syndrome patient undergoing leniolisib treatment

**DOI:** 10.3389/fimmu.2024.1517543

**Published:** 2025-01-13

**Authors:** Francesca Conti, Mattia Moratti, Elena Sabattini, Pier Luigi Zinzani

**Affiliations:** ^1^ Pediatric Unit, IRCCS Azienda Ospedaliero-Universitaria di Bologna, Bologna, Italy; ^2^ Department of Medical and Surgical Sciences (DIMEC), University of Bologna, Bologna, Italy; ^3^ Specialty School of Paediatrics-Alma Mater Studiorum, University of Bologna, Bologna, Italy; ^4^ Department of Systems Medicine, University of Tor Vergata, Rome, Italy; ^5^ Haematopathology Unit, IRCCS Azienda Ospedaliero-Universitaria Di Bologna, Bologna, Italy; ^6^ IRCCS Azienda Ospedaliero-Universitaria di Bologna, Istituto di Ematologia “Seràgnoli”, Bologna, Italy

**Keywords:** activated PI3K delta syndrome (APDS), leniolisib, Hodgkin (cHL), PI3K inhibitor, Hodgkin lymphoma (HL)

## Abstract

Activated PI3K delta syndrome (APDS) is a primary immunodeficiency that is caused by mutations in the PI3K signalling pathway resulting in either gain-of-function or loss-of-function phenotypes of APDS 1 and 2. Malignancy is one of the most serious complications associated with APDS patients, with the most commonly occurring of these being lymphoma, and is the most common cause of death in APDS patients. Management of APDS is complex and variable due to the heterogeneous nature of the disease and ranges from antimicrobial and immunosuppressant agents to haematopoetic stem cell transplantation. More recently, an increasing level of interest has been shown in the use of more targeted agents such as PI3Kδ-specific inhibitors. Here, we provide expert perspective on the suspected causality of a case of lymphoma observed in a 20-year-old female patient who was included in a clinical trial of leniolisib, a PI3K inhibitor.

## Introduction

APDS is a primary immunodeficiency disease caused by hyperactivation of the PI3K signalling pathway, which has an essential role in human immune function (1–3).

Mutations in either the p110δ catalytic subunit or the p85α regulatory subunit give rise to the APDS phenotypes. Gain-of-function mutations in the *PIK3CD* gene give rise to APDS1 and loss-of-function mutations in the *PI3KR1* gene, to APDS2 (1–3). Clinical manifestation of APDS1 and 2 include recurrent infection, non-neoplastic lymphoproliferation, enteropathy and cytopenias, bronchiectasis, neurodevelopmental delay, and growth retardation (4–14, 49).

Malignancy is one of the most serious complications of APDS. In a 2020 systematic review by *Jamee* et al., 31 malignancies were reported in 243 APDS patients (12.8%), with lymphoma being the most common type (89%). Diffuse large B-cell lymphoma (DLBCL) was the most frequent (14/243, 5.7%), followed by classical Hodgkin’s lymphoma (cHL; 9/243, 3.7%) and marginal zone B-cell lymphoma (5/243, 2.0%) (14). These data are supported by additional retrospective studies describing the clinical features of national and international APDS cohorts, including those from the European Society for Immunodeficiencies (ESID) Registry and the United States Immunodeficiency Network (USIDNET), reporting an overall malignant lymphoproliferation of 13% and 12%, respectively, with rates reaching up to 25% in a cohort study by Coulter et al., with non-HL being generally predominant over cHL and associated in many cases with EBV infection (4–13).

Management of APDS is varied due to the heterogeneous nature of the disease and ranges from watch and wait to symptomatic treatment to HSCT depending on severity (15, 16). Antimicrobial agents and immunoglobulin replacement therapy are frequently used to treat and/or prevent recurrent infections; however, they have limited impact on autoimmunity and lymphoproliferation (15, 16). Immunosuppressive agents are therefore the mainstay of treatment to address autoimmunity and lymphoproliferation symptoms with use of corticosteroids having been shown to be effective in treating cytopenias, inflammatory colitis, and renal disease, among others. HSCT is currently the only available treatment with curative potential. Nevertheless, it is usually reserved for the most severe cases due to the invasiveness of such treatment and the paucity of data available regarding its efficacy with only 10%–20% of patients having been treated (15). Despite the myriad treatments available for APDS in the past, survival for individuals appears to be reduced from the average lifespan; a recent comprehensive review of 256 published APDS cases reports a 74% survival rate at 30 years, with the most common cause of death being lymphoma and HSCT (17).

More recently, targeted therapies have been of increasing interest and there is a growing focus on specific inhibitors of PI3Kδ, among which idelalisib and duvelisib have currently been approved by the Food and Drug Administration (FDA) to treat cancers with an expanding interest in clinical trials for immunodeficiency diseases (18–20). However, the existing PI3K inhibitors still have some defects that cannot be ignored, such as adverse effects and drug resistance. Therefore, it is necessary to further develop novel PI3K inhibitors with universality, low toxicity, and high efficiency (21).

There are several clinical trials evaluating the safety and efficacy of PI3Kδ inhibitors in patients with APDS. The first of these with leniolisib (NCT02435173) included a randomised, placebo-controlled phase 3 trial; 31 patients with APDS aged ≥12 years were randomised to receive 70 mg leniolisib or placebo twice daily for 12 weeks. Overall, leniolisib was well tolerated and significantly reduced lymphadenopathy and increased naïve B cells% versus placebo, reflecting a favourable impact on immune dysregulation and deficiency.

The second trial (NCT02859727) is an ongoing single-arm, open-label extension (OLE) of the abovementioned trial to establish the long-term safety of leniolisib in the same cohort (22). In addition, there are further three open-label studies underway in Japanese patients (NCT06249997) and paediatric patients aged 4–11 (NCT05438407) and aged 1–6 (NCT05693129). Leniolisib is now approved by the FDA for the treatment of patients with APDS ≥12 years old. Other development programmes assessing the efficacy and safety of selatalisib and nemiralisib have been halted (23, 24).

## Case presentation

During the ongoing OLE study of leniolisib in 37 patients with APDS, one grade 3 and serious adverse event of classical Hodgkin’s lymphoma leading to leniolisib discontinuation and withdrawal from the study was reported in mid-2022 at extension day (ED) 750.

The affected female participant of Asian descent was a young adult at the time of cHL diagnosis; her medical history included lymphoproliferation and recurrent otosinopulmonary infections starting in early childhood, persistent EBV viremia from late childhood, and obesity from adolescence. She was randomised to placebo in the RCT and started leniolisib 70 mg bid in the OLE in mid-2020. Efficacy parameters (assessed out to ED168/252) showed a good response to leniolisib—these included reduction in lymphadenopathy (71% decrease in the sum of product dimensions of index lymph nodes) and spleen volume (51% decrease) and an increase in the proportion of naïve B cells (out of total B cells) from 44% to 94%. In early 2021 (after ~8.5 months of leniolisib exposure), the proband had a ~2-month treatment gap because of COVID-19-related travel restrictions and international customs hold causing supply issues. Over the following ~15 months, the proband experienced numerous adverse events prior to her eventual cHL diagnosis at ED750; neutropenia and suspected COVID-19 necessitated leniolisib interruption, and chronic fever and neutropenia prompted bone marrow evaluation. The treating physician assessed the cHL as unrelated to leniolisib. Throughout the study, the treatment team had concerns about adherence to the recommended dosing regimen as study drug administration timing was irregular, coincident with the erratic sleep patterns often seen in adolescents/young adults. Following successful cHL therapy, the treating team were keen for the patient to restart leniolisib because of the clinical benefits seen; however, ultimately the decision was taken to proceed to HSCT.

## Non-APDS-related intrinsic Hodgkin’s lymphoma risk

According to a 2022 study by *Huang* et al. evaluating global trends in Hodgkin’s lymphoma, there was an increase in Hodgkin’s lymphoma incidence, especially among females, adolescents, and Asian populations (25). In 2020, 0.4% of worldwide newly reported cancer-related cases were Hodgkin’s lymphoma; although relatively rare, it is the most common cancer in 15–19-year-olds (25).

Male sex, adolescence and young adulthood, a history of EBV infection, HIV/AIDS, autoimmune diseases, obesity, hypertension, pollution exposure, cigarette smoking, family history, and a high socioeconomic status were the main factors significantly associated with Hodgkin’s lymphoma (**Table 1**) (25). In particular, incidence was associated with smoking and obesity in females and with a higher Gross Domestic Product (GDP) per capita, smoking, alcohol intake, and obesity in individuals aged below 50 (25).

**Table 1:** Proband’s lymphoma risk factors and classification according to WHO-HAEM5*.

Histological diagnosis	Viral association	Immune deficiency/dysregulation setting	Non-APDS-related intrinsic risk factors
Stage IV Hodgkin’s lymphoma	EBV-positive viremia	Activated phosphoinositide 3-kinase delta syndrome type 1 (APDS 1)	Ethnicity (Asian) Age (young adulthood) EBV infection (recurrent viremia since late childhood) Weight (obesity) Autoimmune disease (cytopenias) Family history (maternal leukaemia)

WHO-HAEM5, World Health Organization Classification of Haematolymphoid Tumours, Fifth Edition.

## APDS-related Hodgkin’s lymphoma risk

APDS is characterised by immune system dysregulation, specifically an increase in transitional B cells and a decrease in naïve B-cell numbers, accompanied by a decrease in naïve T cells and an increase in T-cell senescence (4–14, 49). T cells play a crucial role in infectious, inflammatory, and neoplastic conditions, where they exert T-cell-mediated cytotoxic activity and support B-cell humoral responses. T-cell exhaustion, senescence, or inactivation, whatever the mechanism, brings along a higher risk of development of malignancies. Given the pivotal role played by the CD8+ cytotoxic T-cell subset in EBV infection control, it is not surprising that EBV-positive lymphoproliferations/lymphoproliferative disorders (EBV+LPD) are frequently observed in immunodeficient settings, including inborn error ones.

Given that the neoplastic cells of cHL (classical Hodgkin’s lymphoma) occurring in immune-competent settings can also integrate EBV, the histologic picture of this lymphoma type in immune-competent and immune-deficient conditions largely overlaps (26, 27), although some features can be more commonly observed in cHL in immune-suppressed patients, such as more frequent expression of B-cell-associated markers by neoplastic cells (26, 28). Indeed, PI3K subunits play a key role in oncogenesis: somatic mutations in p110δ (causative for APDS1) have been found in locations analogous to oncogenic variants in p110α and in patients with non-APDS-related DLBCLs; furthermore, tumour-suppressor p85α mutations (causative for APDS2), leading to p110α hyperactivation, have been found in gliomas and colon, endometrial, and breast cancers (14, 29, 30).

Moreover, the APDS intrinsic tumour risk can be enhanced by viral infection. *Jamee* et al. showed that the frequency of malignancy was higher in patients with a history of (chronic) viral infections than in those without (19.5% vs. 7.4%, p=0.006), in particular, by EBV targeting B cells and representing a reservoir for the virus, which infected almost half of the patients diagnosed with lymphoma (14).

Eventually, APDS-related immune dysregulation, specifically in terms of T-cell senescence and exhaustion resulting in reduced CD8+ T cytotoxicity, dampens prompt viral clearance and favours a persistent inflammatory response promoting herpetic susceptibility, lymphoproliferation, and ineffective tumour surveillance (14, 31).

## Potential for PI3Kδ-inhibitor-related Hodgkin’s lymphoma risk

There are currently no published data supporting a link between cancer development and leniolisib employment, especially as regards Hodgkin’s lymphoma; rather, leniolisib significantly reduced lymphoproliferation in terms of lymphadenopathy and splenomegaly regression, which may reduce the risk for neoplastic degeneration of ADPS-related lymphoproliferative disorders (22, 32, 33). Nonetheless, *Compagno* et al. stated that PI3Kδ inhibition could result in genomic instability favouring lymphoma development as a consequence of activation-induced cytidine deaminase (AID) overexpression in B cells (34).

In fact, idelalisib and duvelisib, both PI3Kδ inhibitors, were found to prompt the expression of AID in B cells, increasing somatic hypermutation and potential oncogenic chromosomal translocation frequency both *in vitro* and *in vivo*, respectively, in human cancer cell-lines, and in mice and patients treated with idelalisib (34).

The dysregulation of AID plays a critical role in the pathophysiology of APDS, particularly affecting B-cell function and antibody diversity. This dysregulation impairs somatic hypermutation and class-switch recombination in B cells, essential processes for generating high-affinity antibodies and diversifying the antibody repertoire (35, 36).

In APDS, these impairments are due to defective, rather than exaggerated, AID expression, leading to elevated serum IgM levels and reduced IgG and IgA levels, which are hallmarks of the disease (35, 36).

So, leniolisib correlation with Hodgkin’s lymphoma remains currently a mere inductive speculation, first of all because leniolisib could normalise AID under-activation, rather than causing its over-activation, in APDS patients, and ultimately AID levels were not evaluated in the #NCT02435173 clinical trial cohort (33, 35, 36).

## Expert perspectives

Below, we try to explore the possible causality assessment between the development of Hodgkin’s lymphoma and the assumption of leniolisib in the proband. These perspectives were initially provided independent of one another to the sponsor of the leniolisib clinical trial programme, who had requested an independent review of the case.

### The immunologist point of view

#### Proband-related Hodgkin’s lymphoma diagnosis and associated risk factors

Referring to the aforementioned considerations, the following are taken into account:

- Malignancy is one of the most serious complications of APDS with a median onset age of 18 years; in particular, lymphoma has been reported in up to 25% of affected patients, with Hodgkin’s lymphoma being the second most frequent lymphoma variant, diagnosed in mid-2022 at the age of 20 years in the proband (4–6, 10, 12, 37).
- The APDS-related tumour risk is increased in patients with a history of viral infection, in particular EBV and CMV (14, 31): in the case in question, lymphadenopathies, appeared since early 2008, resulted to be EBV-positive at some biopsies, with the first positive EBV viremia reported in early 2013, with peaks of more than 4.000 DNA copies/ml in mid-2021 and early/mid-2022.
- APDS-related immune dysregulation encompasses neoplastic degeneration (14, 31, 38): in the proband, immune dysregulation showed an early onset with cytopenias since 6 years of age, an undated history of leukopenia, recurrent otitis media since late 2008, bronchiectasis since early 2009, sinusitis exacerbations since early 2014, asthma since early 2017, immunophenotype imbalance (increased CD8+T-cells% with a reversed CD4/CD8 ratio, reduced CD19+ total, naïve and memory B-cells%, augmented transitional B-cells%) since mid-2017, recurrent fever of unknown origin since early 2019, interstitial lung disease since late 2019, lymphadenopathy, and splenomegaly, all conditioning infectious susceptibility, regarding in particular the upper and lower respiratory tracts and resulting in a persistent inefficient immune system activation allegedly favouring neoplastic evolution.
- According to a 2022 worldwide study, Hodgkin’s lymphoma incidence is increasing, especially among females, youngsters, and Asian populations, all criteria met by the proband, a 20-year-old woman of Middle Eastern origin; moreover, a history of EBV infection, autoimmune diseases, and obesity constitute further features detected in the patient associated with Hodgkin’s lymphoma development (25).
- In literature, there is to date no evidence-based mention about a possible link existing between leniolisib employment and Hodgkin’s lymphoma but only a pure inductive hypothesis regarding the potential oncogenic role of PI3Kδ inhibitor-related AID over-expression; rather, leniolisib-related significant regression of lymphadenopathies and splenomegaly could reduce the risk for neoplastic degeneration. In this context, it is worth considering that the patient discontinued therapy three times over the 2 years of treatment (from February 2021 to May 2021, for 63 days, with subsequent EBV reactivation characterised by recrudescence of febrile lymphadenopathies, splenomegaly, and high load EBV viremia; in end-February/March 2022 for 10 days for a viral infection with febrile neutropenia and anaemia, and in June 2022 for symptomatic SARS-CoV-2 infection), and this probably negatively affected the disease course.

It would therefore not be reasonable to attribute the development of Hodgkin’s lymphoma to leniolisib.

### The pathologist point of view

From a pathologic point of view, the recently published World Health Organisation and International Consensus Conference classifications of lymphoid tumours recognise several histologic patterns of EBV+LPD in immunodeficient settings, including cHL (27, 30). Regarding the proband, a 20-year-old woman of Middle Eastern origin, pathologic features of the deficient immune status were present in the several lymph node biopsies that were performed over the years: in fact, although being reported as negative for an EBV+LPD (either of polymorphic or monomorphic types), EBV+ cells were sometimes recorded. This phenomenon is not infrequent in tissues taken from patients whose T-cell control against the latent EBV infection is impaired (39). If the condition persists, the intrinsic oncogenic power of the virus might take over the antagonist capacity of the immune system and lead to the malignant evolution of the EBV+ cell.

From the histologic description of the bone marrow biopsy (performed early/mid-2022) for cytopenia, no clear-cut hints of malignancy were reported. A diffuse atypical lymph histiocytic proliferation with rare scattered large, atypical cells, EBV negative, was reported, which could not reliably confirm or exclude the presence of a lymphoma. Moreover, presence of eosinophils (typical of cHL) was not mentioned. In no case can this type of infiltration be reliably related to a drug reaction.

Compagno et al. in 2017 suggested a possible role of PI3Kδ inhibition in lymphoma development as the result of the B-cell genomic instability induced by the drug-related overexpression of AID; the latter protein has a role in the process of somatic hypermutation in germinal centres (34). Although a drug-induced instable genomic background cannot be ruled out as a potential favouring element, it neither necessarily relates to Hodgkin lymphoma nor does it explain the temporal relationship between EBV increase and Hodgkin lymphoma development.

In conclusion, given 1) the established relationship between cHL and EBV, 2) the young age of the patient (typical of cHL onset), 3) the observed presence of EBV-positive cells in previous biopsies tissues, 3) the episodes of high and persistent EBV viremia, also close to the lymphoma onset, 4) the absence of reported similar events in the two available clinical trials with PI3K inhibitors (www.clinicaltrials.gov as #NCT02859727 and #NCT02435173), and the 5) possible contribution of a 63-day-long leniolisib interruption, within the primary immune-deficient setting the proband has been suffering from, one would tend to favour the inborn error immunity disease as the major potential cause of the development of cHL, which is likely unrelated to the drug administration.

### The haematologist point of view

Given the complexity and importance of the PI3K-Akt signalling cascade, pharmacological PI3K inhibition results in the following (40, 41):

- inhibition of cytokine signalling from the microenvironment through chemokine-mediated and adhesion molecule-mediated pro-survival signals, by means of mechanisms mostly relying on p110δ;
- functional impairment of CD8+ T lymphocytes and reduction in the ability of CD4+ T lymphocytes to proliferate, expand, and differentiate into helper T-cell subsets, as a consequence of p110δ inhibition, on which signals from the T-cell receptor converge;
- reduced chemotaxis of activated CD4+ and CD8+ T cells via the stimulation of chemokine receptors, as a result of p110γ blockade;
- decreased T-regulatory lymphocyte functions, which consequently counterbalance the impaired T-CD4+ and CD8+ function;
- possible enhancement of direct anti-tumour activity.

These effects appear of particular importance in lymphoproliferative malignancies, as lack of p110δ and p110γ in knock-out mice was associated with impaired immune response and B-cell development, and mutations in the *PIK3CD* gene (encoding p110δ) have been associated with increased cancer susceptibility and occurrence of B-cell lymphomas (42, 43).

PI3K inhibitors have been widely approved in the treatment of indolent non-Hodgkin lymphomas, mainly Follicular lymphoma (FL), in case of disease relapse or refractoriness to anti-CD20 agents, alkylators, or purine analogues. Their efficacy has been confirmed in phase 2 and 3 clinical trials, and potential combinations with monoclonal antibodies, chemotherapy, or targeted agents are under investigation (44–48). Treatment-emergent toxicities are still an issue, given that a significant proportion of patients experience extra-haematologic—and prevalently immune-mediated—adverse effects and display enhanced infectious morbidity. Newer PI3K inhibitors, with higher selectivity towards the PI3Kδ isoform or with a mechanism of action that combines the inhibition of multiple substrates, may display an enhanced on-target effect with a concomitant reduction in adverse events.

On the basis of these data, it is reasonable to think that the onset of Hodgkin lymphoma is unrelated to leniolisib.

## Conclusion

In conclusion, evidence presented in this expert perspective paper strongly suggests that attributing the development of Hodgkin’s lymphoma in the proband to leniolisib intake is not substantiated. The following key points underscore this assumption:

1. There is a critical need for effective target therapies to manage APDS; according to current literature, PI3Kδ inhibitors mitigate associated comorbidities, in particular non-clonal lymphoproliferation, allegedly reducing neoplastic degeneration to lymphoma.
2. The onset of lymphoma in the patient appears to be closely linked to the underlying APDS rather than leniolisib. Multiple factors, including immune dysregulation, persistent EBV infection, and other predisposing genetic factors, likely contribute to lymphomagenesis.
3. Long-term follow-up studies indicate a favourable safety profile for leniolisib, with no clear evidence of an increased risk of Hodgkin’s lymphoma associated with its use. This further supports the notion that the lymphoma development in this case is not directly linked to leniolisib.
4. While existing data suggest no causal link between leniolisib and Hodgkin’s lymphoma, ongoing trials and larger cohort studies are necessary to comprehensively assess the relationship between leniolisib use and lymphoma risk, particularly across different age groups.

## Data Availability

The original contributions presented in the study are included in the article/supplementary material. Further inquiries can be directed to the corresponding author.
